# Spatial Distribution of Neuropathology and Neuroinflammation Elucidate the Biomechanics of Fluid Percussion Injury

**DOI:** 10.1089/neur.2020.0046

**Published:** 2021-02-08

**Authors:** Joshua A. Beitchman, Jonathan Lifshitz, Neil G. Harris, Theresa Currier Thomas, Audrey D. Lafrenaye, Anders Hånell, C. Edward Dixon, John T. Povlishock, Rachel K. Rowe

**Affiliations:** ^1^ BARROW Neurological Institute at Phoenix Children's Hospital, Phoenix, Arizona, USA.; ^2^ Child Health, University of Arizona College of Medicine–Phoenix, Phoenix, Arizona, USA.; ^3^ Midwestern University, Glendale, Arizona, USA.; ^4^ Arizona State University, Tempe, Arizona, USA.; ^5^ Phoenix VA Health Care System, Phoenix, Arizona, USA.; ^6^ UCLA Brain Injury Research Center, Department of Neurosurgery, and Intellectual Development and Disabilities Research Center, David Geffen School of Medicine, University of California at Los Angeles, Los Angeles, California, USA.; ^7^ University of Pittsburgh, Pittsburgh, Pennsylvania, USA.; ^8^ Virginia Commonwealth University, Richmond, Virginia, USA.; ^9^ Uppsala University Hospital, Uppsala, Sweden.

**Keywords:** biomechanics, FPI, histopathology, temporal ridge

## Abstract

Diffuse brain injury is better described as multi-focal, where pathology can be found adjacent to seemingly uninjured neural tissue. In experimental diffuse brain injury, pathology and pathophysiology have been reported far more lateral than predicted by the impact site. We hypothesized that local thickening of the rodent skull at the temporal ridges serves to focus the intracranial mechanical forces experienced during brain injury and generate predictable pathology. We demonstrated local thickening of the skull at the temporal ridges using contour analysis on magnetic resonance imaging. After diffuse brain injury induced by midline fluid percussion injury (mFPI), pathological foci along the anterior-posterior length of cortex under the temporal ridges were evident acutely (1, 2, and 7 days) and chronically (28 days) post-injury by deposition of argyophilic reaction product. Area CA3 of the hippocampus and lateral nuclei of the thalamus showed pathological change, suggesting that mechanical forces to or from the temporal ridges shear subcortical regions. A proposed model of mFPI biomechanics suggests that injury force vectors reflect off the skull base and radiate toward the temporal ridge, thereby injuring ventral thalamus, dorsolateral hippocampus, and sensorimotor cortex. Surgically thinning the temporal ridge before injury reduced injury-induced inflammation in the sensorimotor cortex. These data build evidence for temporal ridges of the rodent skull to contribute to the observed pathology, whether by focusing extracranial forces to enter the cranium or intracranial forces to escape the cranium. Pre-clinical investigations can take advantage of the predicted pathology to explore injury mechanisms and treatment efficacy.

## Highlights

- The temporal ridge is 75% thicker than the adjacent skull of the rodent.
- Experimental diffuse TBI neuropathology occurs beneath the length of the temporal ridge.
- Neuropathology encompasses sensorimotor cortex, somatosensory thalamus, and dorsolateral hippocampus.
- Proposed mechanism of biomechanical injury forces include the temporal ridge.

## Introduction

Clinical and experimental traumatic brain injury (TBI) involve a primary injury and subsequent pathophysiologies that dismantle, repair, and regenerate circuits in the brain.^1–3^ In response to the mechanical injury, adaptive repair and regeneration fail to reconstitute the original neuronal circuits, leaving a miswired brain and neurological impairments that decrease quality of life. Clinically, this process is relevant to veterans, athletes, survivors of interpersonal violence, children, and the elderly who experience one or more TBIs.^4,5^ We and others apply experimental models of TBI to study the acute and chronic events associated with this disease and use these models to develop therapeutic interventions.

Various models of experimental TBI have been developed, where fluid percussion injury (FPI) is one of the best characterized animal models of TBI.^1,6–9^ Specifically, the midline FPI (mFPI) model produces a diffuse, concussive-like TBI in rodents, whereas lateral FPI (lFPI) produces a mixed focal and diffuse injury.^1,8,10–12^ To this end, the primary FPI neuropathology is diffuse axonal injury, rather than overt cell death. Clinical relevance of these injuries includes a transient suppression of neurological reflexes and acute motor deficits.^6,7,11,13–29^ In the more chronic phases of injury, evidence of somatic, cognitive, and affective symptoms emerges. Somatic morbidity includes a hypersensitivity to facial whisker stimulation, similar to agitation in people.^30–36^ Cognitive performance is degraded in short, long, and working memory, using various cognitive testing modalities.^11,33,37–47^ Affective symptoms are identified by disruption of circulating hormone levels and responses in anxiety tests.^11,48–50^ Thus, FPI affords investigations into the development and maintenance of cognitive, somatic, and affective morbidities, which parallel clinical impairments.

Despite the broad implementation of the FPI model in the neurotrauma field, biomechanical models fail to accurately explain the resultant distribution of pathology.^51–58^ In the initial implementation of FPI in rodents, Dixon and colleagues describe that this technique does “*not attempt to reproduce rapid acceleration-deceleration of the head…. Rather, fluid-percussion brain injury successfully produces graded levels of injury associated with predictable neurologic, physiologic and histologic changes that are comparable to those observed in human brain injury*.”^15^ In an approach to observe the mechanical forces of injury on the brain, Dixon and colleagues conducted high-speed cineradiographic studies of the brain inside the skull over the 15 msec of mFPI injury.^15^ In this way, the primary injury was first characterized as “*intracranial fluid movement … by rapid radial movement within the epidural space …. suggesting that the image of the indentation acutely may have been caused by lateral fluid displacement following the curvature of the skull*.”^15^

Thereafter, subsequent publications comment on the resultant pathology. Hicks and colleagues reported that “*it is interesting to note that the primary site of cortical damage is ventrolateral, rather than directly underneath the impact site.*”^59^ These researchers continue to explain this phenomenon as a consequence of biomechanical forces on selectively vulnerable tissue, based on regional or cellular cytoarchitecture. Over the 30+ years of research using FPI in rodents, curious pathology has been reported as focal damage far more lateral than predicted by the location of the applied mechanical forces of injury and remains an enigma. For the midline variant of FPI, the core pathologies are identified millimeters more lateral from the injury site. Further, cortical pathology does not necessarily align with cytoarchitectural landmarks, as posited by Hicks and colleagues.^59^ The curious pathology occurs far lateral from the injury site in other non-focal TBI models.^60^

In this communication, we reference the range of pathologies occurring far lateral from the injury site to include, but in no way are limited to, blood–brain barrier disruption, axotomy, plasma membrane permeability, and cell death. Upon re-examination, we recognized that the curious pathology of diffuse TBI was tracked beneath the temporal ridge of the skull. We make a case for differential thickness along the rodent skull as a contributing factor to the direction of biomechanical forces of diffuse brain injury, in addition to inherent properties of the tissue. Thus, we hypothesized that a local thickening of the rodent skull at the temporal ridges serves to focus mechanical forces of brain injury and generate predictable pathology in line with the temporal ridges.

## Methods

### Compendium of experimental traumatic brain injury publications

A compendium of literature was assembled by using experimental brain injury publications to identify low-power photomicrographs that include primary sites of pathology as photomicrographs or schematics in complete or hemispheric coronal sections. All figures appearing in the hard-copy publications of the *Journal of Neurotrauma* (1987–2010) were screened manually. Relevant flagged images were then searched on https://images.google.com for higher-resolution histological or radiographical coronal images of brain-injured rodents.

### Animals

Animal work was conducted using 8- to 12-week-old male Sprague-Dawley rats. Rats were pair-housed in a normal 12-h light/dark cycle with food and water available *ad libitum*. All practices were conducted in accordance with the guidelines established by the internal Institutional Animal Care and Use Committee and the National Institutes of Health (NIH) Guidelines for the Care and Use of Laboratory Animals. Studies are reported following the Animal Research: Reporting *In Vivo* Experiments (ARRIVE) guidelines.^61^ Randomization of animals was achieved by assigning animals to treatment groups before initiation of the study. Animals were evaluated daily for 3 days post-operatively. Pre-determined exclusion criteria included post-operative weight loss >15% of pre-surgical weight. No rats were excluded from this study.

### Flesh-eating beetles

Skulls from 8- to 12-week-old naïve rats were prepared by dermestid beetles (*dermestes maculatus*). Rat heads were skinned, hung to dry, and then placed in glass jars. Within 10–14 days, carrion was cleaned from skulls by the beetle larvae. Skulls were further cleaned with bleach water and air-dried. Photographs were taken of complete rat skulls and rat skulls that were cut in the coronal plane with a hacksaw (exposed surfaces were marked with permanent marker to increase contrast). Measurements were taken along the circumference of coronal sections of the calvarium using calipers on multiple rostral-caudal sections, focusing on the medial-lateral midpoint of the parietal bone and the temporal ridge.

### Magnetic resonance imaging of the rat head

A cohort of naïve 8- to 12-week-old rats was anatomically imaged by magnetic resonance imaging (MRI) to visualize the relationship between the brain, skull, and musculature. All data were acquired on a 7 Tesla (T) spectrometer (Oxford Instruments, Oxford, UK) controlled by a Bruker Biospec console (Bruker Biospin MRI Inc, Billerica, MA). The rat was anesthetized with isoflurane (4% induction, 1.5% maintenance, vaporized in oxygen) positioned in a purpose-build plexiglass cradle using a bite-bar and ear bars.

Data were acquired using a ^1^H radiofrequency (RF) volume resonator in transmit-only mode and a pulse-decoupled receive-only surface RF coil placed over the head. Image acquisition was performed using a two-dimensional, rapid acquisition with relaxation enhancement (RARE) pulse sequence using a 35 × 35 mm field of view encoded in a 128 × 128 data matrix, with 50 coronal image slices 0.5 mm thick, resulting in a resolution of 234 × 234 × 750 μm. The following imaging parameters were used: 6-sec repetition time, 56-msec echo time, 50-kHz bandwidth, four averages per phase-encoding increment, and RARE factor 8. Data were Fourier transformed into 16-bit signed integer spatial data and then regrouped into compressed NIFTI format. Image stacks were evaluated using the Volume Viewer 1.31 plugin on NIH Image. Images were rotated, segmented, and pseudocolored to represent relationships between the brain, skull, and musculature of naïve rats with respect to the temporal ridges of the skull.

### Midline fluid percussion injury

Adult male Sprague-Dawley rats were subjected to mFPI, consistent with methods described previously.^10,22,62,63^ Briefly, rats were anesthetized with 5% isoflurane in 100% O_2_ and maintained at 2%/100% O_2_ by nose cone. During surgery, a 4.8-mm circular craniotomy was performed (centered on the sagittal suture midway between bregma and lambda) without disrupting the underlying dura or superior sagittal sinus. An injury cap was fabricated from the female portion of a Luer-Loc needle hub. A skull screw was secured in a 1-mm hand-drilled hole into the right frontal bone. The injury hub was affixed over the craniotomy, and the incision was sutured at the anterior and posterior edges. Animals were returned to a warmed holding cage and monitored until ambulatory (∼60–90 min).

For injury induction, animals were reanesthetized with 5% isoflurane. The dura was inspected through the injury-hub assembly for debris, which was then filled with normal saline and attached to the male end of the fluid percussion device (Custom Design and Fabrication; Virginia Commonwealth University, Richmond, VA). An injury of moderate severity (2.0–2.1 atm; 5- to 8-min righting reflex time) was administered by releasing the pendulum onto the fluid-filled cylinder, as reflexive responses returned. Animals were monitored for presence of a forearm fencing response and the return of the righting reflex as indicators of injury severity.^22^ After injury, the injury hub assembly was removed *en bloc*, integrity of the dura was observed, and the incision was stapled. After recovery of the righting reflex, animals were placed in a warmed holding cage before being returned to their home cages. Adequate measures were taken to minimize pain or discomfort.

### Amino-cupric silver technique

At 1, 2, 7, and 28 days post-injury (DPI), brain-injured rats (*n* = 3 per time point) were overdosed with sodium pentobarbital (200 mg/kg intraperitoneally) and transcardially perfused with 0.9% sodium chloride, followed by a fixative solution containing 4% paraformaldehyde. After decapitation, the heads were stored in a fixative solution containing 15% sucrose for 24 h, after which brains were removed, placed in fresh fixative, and shipped for histological processing to Neuroscience Associates Inc. (Knoxville, TN). Rat brains were embedded into a single gelatin block (Multiblock^®^ Technology; Neuroscience Associates).

Individual cryosections containing all rat brains were mounted and stained with the de Olmos aminocupric silver technique according to proprietary protocols (Neuroscience Associates), counterstained with Neutral Red, and then cover-slipped. Every sixth section from the anterior commissure through the substantia nigra was imaged at 1.25 × , masked from the background, and overlaid on the remaining sections from the same brain to show extent of neuropathology throughout the brain. Uninjured sham animals were included in these cohorts and have been published with regard to detailed analysis of region-specific neuropathology.^48–50,64–69^ Sections at individual bregma levels were selected to show the primary motor cortex, CA3 hippocampus, and ventral posterior thalamus based on anatomical coordinates using the Watson and Paxinos Rat Brain Atlas.

### Shaved temporal ridge of the skull and immunohistochemistry

Similar to the mFPI surgical procedure describe above, a new cohort of rats (*n* = 9) was prepared for injury induction. In addition to the procedures above, none (*n* = 3), the rat's anatomical left (*n* = 3), or both (*n* = 3) temporal ridge(s) of the skull were shaved by manual scraping to approximate the thickness of the calvarium. Rats were randomly assigned to have the temporal ridges shaved and administered a moderate FPI. At 7 DPI, rats were given an overdose of sodium pentobarbital and transcardially perfused with 4% paraformaldehyde/phosphate-buffered saline. Brains were cryosectioned at 20 μm, wet-mounted onto gelatinized glass slides, and stained for ionized calcium-binding adaptor molecule 1 (Iba-1; rabbit primary antibody IBA-1, 1:1000, Item #0199-19741; Wako Chemicals, Richmond, VA; biotinylated horse antirabbit secondary antibody, 1:250; Vector Laboratories, Burlingame, CA) with diaminobenzidine visualization. Sections depicting individual bregma levels were chosen to present discrete anatomical locations, including the primary motor, CA3 hippocampus, and ventral posterior thalamus, based on anatomical coordinates using the Watson and Paxinos Rat Brain Atlas. Once identified, immunostained slides were imaged (Olympus AX80 Automatic Research microscope with attached DP70 digital camera; Olympus Corporation, Tokyo, Japan).

### Statistical analysis

Data were organized using Microsoft Excel^®^ (Microsoft Corporation, Redmond, WA) and analyzed using Prism^®^ software (Graphpad Software, Inc, La Jolla, CA). Data points collected bilaterally (e.g., thickness of the skull) were averaged to represent a single animal before comparison. A Student's two-tailed *t*-test was used to compare values between groups, with significance defined at *p* < 0.05.

## Results

### Peer-reviewed literature identified traumatic brain injury pathology in cortex beneath the temporal ridge

Cortical pathology beneath the temporal ridge after experimental TBI, particularly FPI in its many variations, has been identified across multiple laboratories over at least a decade. In Table 1, we list 46 publications between the years of 1987 and 2010 with a low-power micrograph or summary schematic of pathology localized under the temporal ridge induced by diffuse or mixed-model brain injury.

**Table 1.** Articles Identifying Pathology along the Temporal Ridge in Rodents

Year	Author	Author	Figure(s)^^*^^	Outcome measure
1987	McIntosh et al.	Traumatic brain injury in the rat: alterations in brain lactate and pH as characterized by 1H and 31P nuclear magnetic resonance	2, 8	Evans Blue extravasation, vulnerable brain region analysis
1987	McIntosh et al.	Traumatic brain injury in the rat: characterization of a midline fluid percussion model	6	Subcortical hemorrhage
1988	McIntosh et al.	Magnesium deficiency exacerbates and pre-treatment improves outcome after traumatic brain injury in rats: 31P magnetic resonance spectroscopy and behavioral studies	1	Evans Blue extravasation
1989	Cortez et al.	Experimental fluid percussion brain injury: vascular disruption and neuronal and glial alterations	4	Evans Blue extravasation
1989	McIntosh et al.	Traumatic brain injury in the rat: characterization of a lateral fluid percussion model	8	Evans Blue extravasation
1990	McIntosh et al.	Effect of non-competitive blockade of N-methyl-d-aspartate receptors on the neurochemical sequelae of experimental brain injury	1	Evans Blue extravasation
1991	Hovda et al.	Diffuse prolonged depression of cerebral oxidative metabolism after concussive brain injury in the rat: a cytochrome oxidase histochemistry study	1	Cytochrome oxidase histochemistry
1991	Yoshino et al.	Dynamic changes in local cerebral glucose utilization after cerebral conclusion in rats: evidence of a hypermetabolic and subsequent hypometabolic state	1	2-Deoxyglucose for glucose metabolic rate
1992	Hovda et al.	Secondary injury and acidosis	5	2-Deoxyglucose for glucose metabolic rate
1992	Soares et al.	Development of prolonged focal cerebral edema and regional cation changes after experimental brain injury in the rat	1	Vulnerable brain region analysis
1993	Hicks et al.	Mild experimental brain injury in the rat induces cognitive deficits associated with regional neuronal loss in the hippocampus.	2	IgG extravasation
1993	Schmidt et al.	Regional patterns of BBB breakdown after central and lateral fluid percussion injury in rodents	6	Biotinylated dextran amine for BBB breakdown
1993	Toulmond et al.	Biochemical and histological alterations induced by fluid percussion brain injury in the rat	6	Benzodiazepine binding for a neuronal marker
1993	Toulmond et al.	Prevention by eliprodil (SL 82.0715) of traumatic brain damage in the rat; existence of a large (18 h) therapeutic window	1	Hematoxylin and eosin
1994	Dietrich et al.	Widespread metabolic depression and reduced somatosensory circuit activation after traumatic brain injury in rats	2	2-Deoxyglucose for glucose metabolic rate
1995	Delahunty et al.	Differential consequences of lateral and central fluid percussion brain injury on receptor coupling in rat hippocampus	4, 5, 6	Cresyl violet
1995	Hicks et al.	Temporal response and effects of excitatory amino acid antagonism on microtubule-associated protein 2 immunoreactivity after experimental brain injury in rats	3	Microtubule-associated protein immunohistochemistry
1995	Rink et al.	Evidence of apoptotic cell death after experimental traumatic brain injury in the rat	2	TUNEL^+^ stain
1995	Soares et al.	Inflammatory leukocytic recruitment and diffuse neuronal degeneration are separate pathological processes resulting from traumatic brain injury	2	Cresyl violet
1995	Soares et al.	Fetal hippocampal transplants attenuate CA3 pyramidal cell death resulting from fluid percussion brain injury in the rat	2	Cresyl violet
1996	Hicks et al.	Temporal and spatial characterization of neuronal injury after lateral fluid percussion brain injury in the rat	1	Acid fuchsin, silver stain
1996	Saatman et al.	Prolonged calpain-mediated spectrin breakdown occurs regionally after experimental brain injury in the rat.	2	Calpain-mediated spectrin breakdown immunohistochemistry
1997	Bareyre et al.	Time course of cerebral edema after traumatic brain injury in rats: effects of riluzole and mannitol	1	Vulnerable brain region analysis
1997	Iwamoto et al.	Investigation of morphological change of lateral and midline fluid percussion injury in rats, using magnetic resonance imaging	1	Magnetic resonance imaging
1997	Perri et al.	Metabolic quantification of lesion volume after experimental traumatic brain injury in the rat	1	TTC for succinate dehydrogenase activity
1997	Smith et al.	Progressive atrophy and neuron death for 1 year after brain trauma in the rat	1	Cresyl violet
1998	Conti et al.	Experimental brain injury induces regionally distinct apoptosis during the acute and delayed post-traumatic period.	2	TUNEL^+^ stain
1998	Hulsebosch et al.	Traumatic brain injury in rats results in increased expression of Gap-43 that correlates with behavioral recovery.	2	Growth-associated protein 43 immunohistochemistry
1998	Murakami et al.	Experimental brain injury induces expression of amyloid precursor protein, which may be related to neuronal loss in the hippocampus.	4	Hematoxylin and eosin
1998	Pierce et al.	Enduring cognitive, neurobehavioral, and histopathological changes persist for up to 1 year after severe. experimental brain injury in rats	5	Cresyl violet
1999	Di et al.	Fluid percussion brain injury exacerbates glutamate-induced focal damage in the rat.	1	Hematoxylin and eosin
1999	Hill-Felberg et al.	Concurrent loss and proliferation of astrocytes after lateral fluid percussion brain injury in the adult rat	4	Proliferating cell nuclear antigen–positive cells for astrocytes, glial fibrillary acidic protein for astrocytes
2000	Matsushita et al.	Real-time monitoring of glutamate after fluid percussion brain injury with hypoxia in the rat	4	TTC for succinate dehydrogenase activity
2000	Passineau et al.	Chronic metabolic sequelae of traumatic brain injury: prolonged suppression of somatosensory activation	1, 2	2-Deoxyglucose for glucose metabolic rate
2001	Harris et al.	Traumatic brain injury–induced changes in gene expression and functional activity of mitochondrial cytochrome c oxidase	5	Cytochrome oxidase enzyme histochemistry
2001	Sato et al.	Neuronal injury and loss after traumatic brain injury: time course and regional variability	1	Vulnerable brain region analysis
2001	Vink et al.	Small shifts in craniotomy position in the lateral fluid percussion injury model are associated with differential lesion development.	1	Magnetic resonance imaging
2002	Bramlett et al.	Quantitative structural changes in white and gray matter 1 year after traumatic brain injury in rats	2	Hematoxylin and eosin
2004	Cernak et al.	The pathobiology of moderate diffuse traumatic brain injury as identified using a new experimental model of injury in rats	7	Hematoxylin and eosin
2004	Hallam et al.	Comparison of behavioral deficits and acute neuronal degeneration in rat lateral fluid percussion and weight-drop brain injury models	3	FluoroJade staining
2004	Singleton et al.	Identification and characterization of heterogeneous neuronal injury and death in regions of diffuse brain injury: evidence for multiple independent injury phenotypes	5	Fluorescent dextrans
2005	Schültke et al.	Neuroprotection after fluid percussion brain trauma: a pilot study using quercetin	2	Luxol fast blue/cresyl violet
2005	Van Putten et al.	Diffusion-weighted imaging of edema after traumatic brain injury in rats: effects of secondary hypoxia	3	Diffusion-weighted imaging
2009	Lotocki et al.	Alterations in BBB permeability to large and small molecules and leukocyte accumulation after traumatic brain injury: effects of post-traumatic hypothermia	5	Biotinylated dextran amine for BBB breakdown
2010	Hayward et al.	Association of chronic vascular changes with functional outcome after traumatic brain injury in rats	2	Cresyl violet, magnetic resonance imaging
2010	Yu et al.	Glial cell–mediated deterioration and repair of the nervous system after traumatic brain injury in a rat model as assessed by positron emission tomography	1	Positron emission tomography

^^*^^Figure number within the cited article.

BBB, blood–brain barrier; IgG, immunoglobulin G; TTC, 2,3,5-triphenyltetrazolium chloride; TUNEL^+^, terminal deoxynucleotidyl transferase dUTP nick end labeling positive.

### Rat skull is thicker at the temporal ridge

**FIG. 1.**
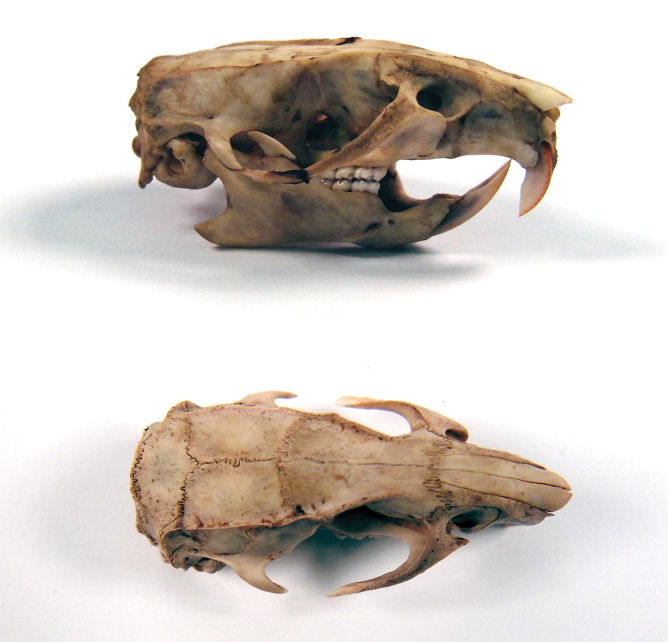
Rat heads were cleaned by flesh-eating beetle larvae until the skull was clear of all carrion. By gaining an anatomical perspective of the adult rat skull, the temporal ridge clearly protrudes on either side, indicating the increased bone mass in this region.

**FIG. 2.**
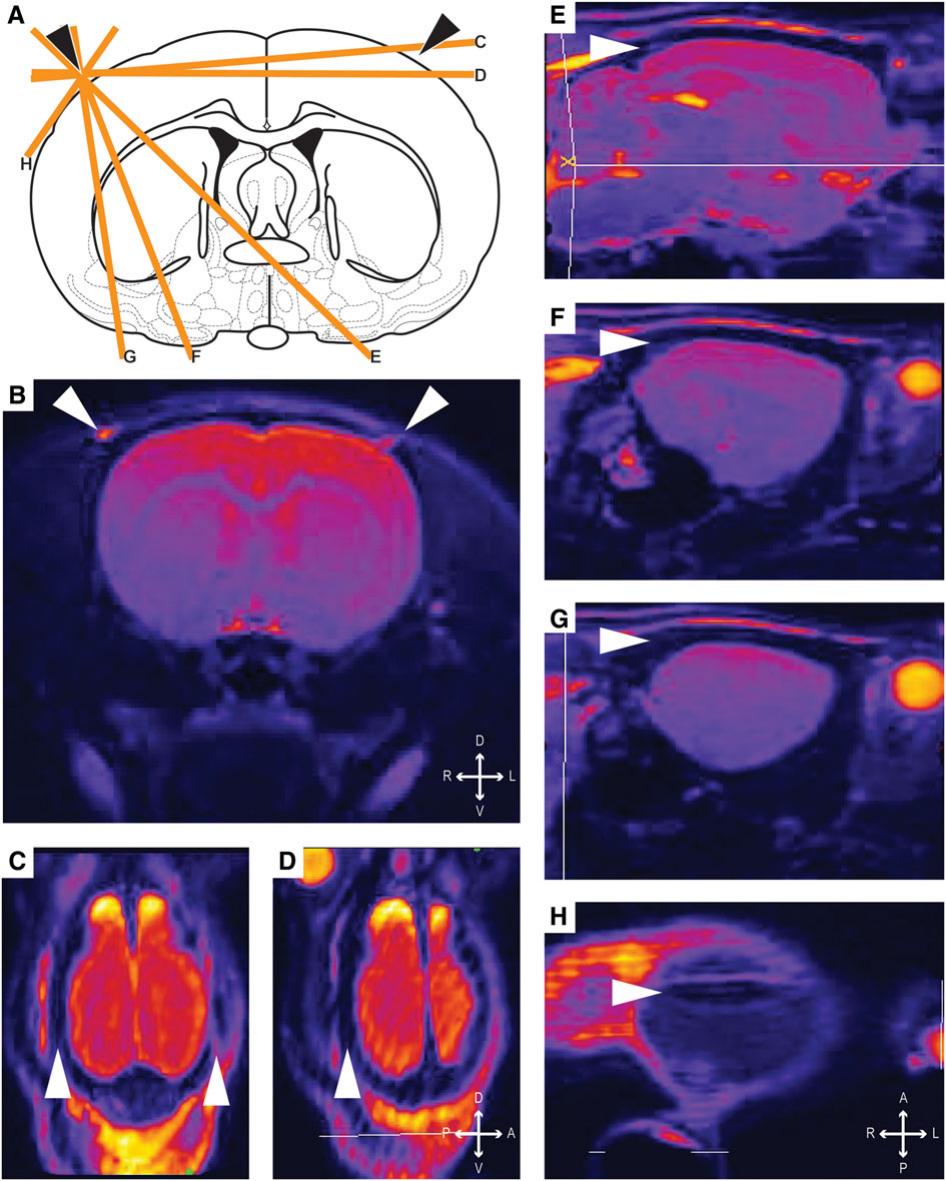
Oblique sections of naïve rat skull using a 7T MRI demonstrated the thickening of the rat skull along the temporal ridges. Coronal schematic (**A**) and section (**B**) present the conventional view of the rat brain. Oblique sections (**C**–**H**) are identified on the schematic at cross through the space beneath the temporal ridge. Temporal ridges are identified with the solid arrow head. MRI, magnetic resonance imaging; T, Tesla.

**FIG. 3.**
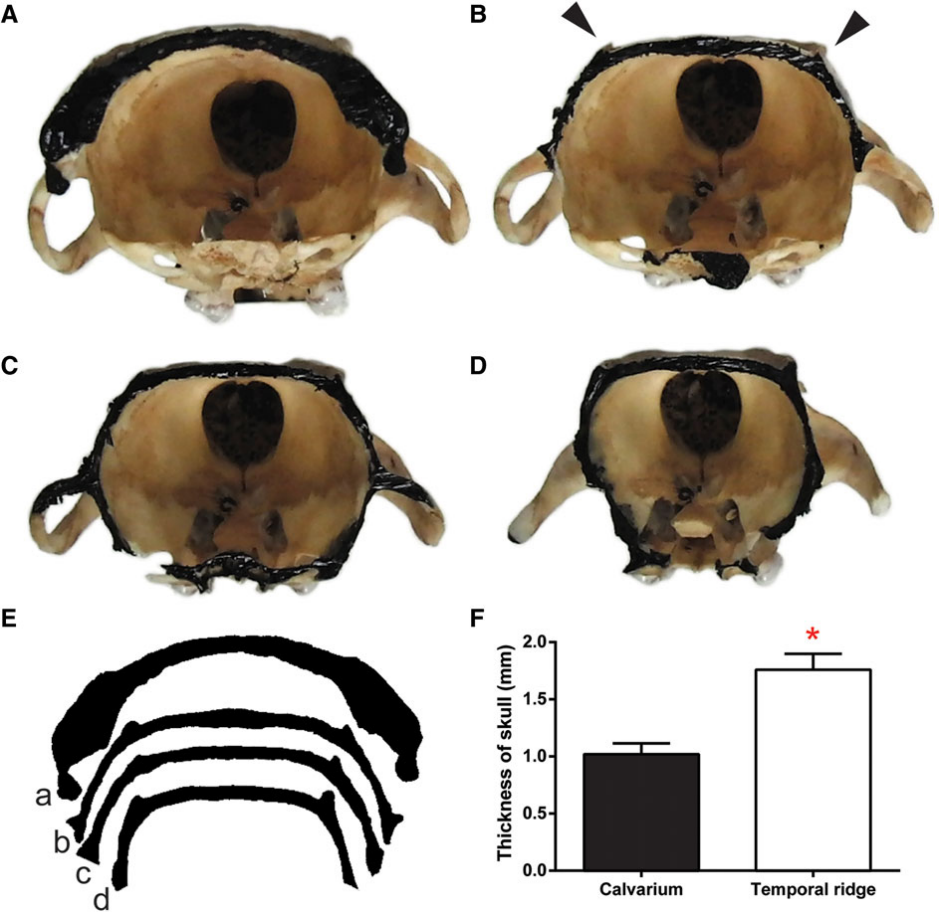
Local thickening of temporal ridges is shown by contour analysis of coronal sections of rat skull. (**A**–**D**) Photographs of rat skull sections are enhanced by black ink on the sectioned edge. Solid arrowheads identify the temporal ridge. (**E**) Digitized outlines of the sectioned edges show the contour changes of the rat skull from posterior (a) to anterior (d). (**F**) Measurements were taken from coronal sections of rats to compare thickness of calvarium to the temporal ridge, where the temporal ridge was 75% thicker.

Naïve rat skulls were cleaned of all tissue using dermestid beetle larvae. Prominence of temporal ridges on the dorsal surface of the skull were evident (Fig. 1). Subsequent *in vivo* imaging was undertaken to demonstrate the relationship between shapes of the skull and brain. Oblique sections of a 7T MRI in naïve rats were prepared to visualize skull thickness with respect to the brain. Coronal (Fig. 2A,B), horizontal (Fig. 2C,D), and oblique sagittal (Fig. 2E–H) slices were all registered to pass through the temporal ridge of the skull (evident in black). Note that thickness of the temporal ridge extended along the anterior to posterior length of the skull (Fig. 2C). The internal surface of the bone contacting the dura and brain is a smooth surface devoid of macrostructure. To confirm imaging results, coronal sections of rat skulls were taken from rostral to caudal, and contour analysis was performed (Fig. 3A–E). Measurements were taken along the temporal ridge and calvarium (*n* = 4) of rat skulls (Fig. 3F). The temporal ridge was found to be 75% thicker than the calvarium (*t* = 4.36; *p* < 0.01).

### Fluid percussion injury–induced argyrophilic neuropathology under the temporal ridge

**FIG. 4.**
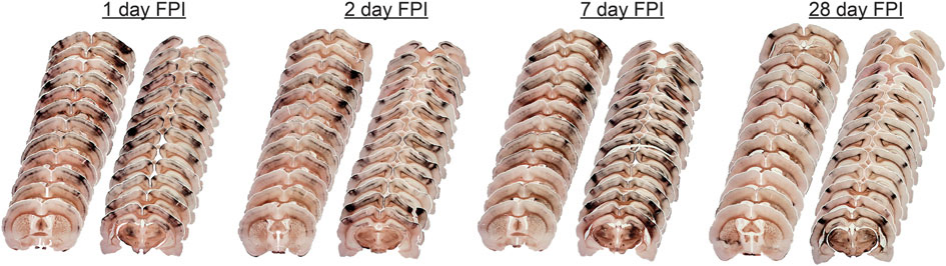
Histological sections of diffuse brain-injured rats are aligned rostral to caudal at 1, 2, 7, and 28 days after midline fluid percussion injury (FPI). Neuropathology was identified by hyperintense deposition of argyrophilic reaction product (amino-cupric silver histochemical technique; black) and occurred primarily along the rostral to caudal extent of sensorimotor cortex. Neuropathology appears to accumulate over 7 days post-injury and mostly subsides by 28 days post-injury. Neuropathology may be diffuse and inconsistent between hemispheres along the rostral-caudal extent of the brain.

Rats were diffuse-brain injured by mFPI and then survived to either 1, 2, 7, or 28 DPI. Brains were then collected, sectioned rostral to caudal**,** and stained with silver to identify regions of neuropathology that develop after diffuse brain injury. Darker (black) stained regions on sections identify hyperintense deposition of argyrophilic reaction product (Fig. 4). Uninjured sham animals were included in these cohorts and have been published along with more detailed analysis for region-specific neuropathology.^48–50,64–69^

**FIG. 5.**
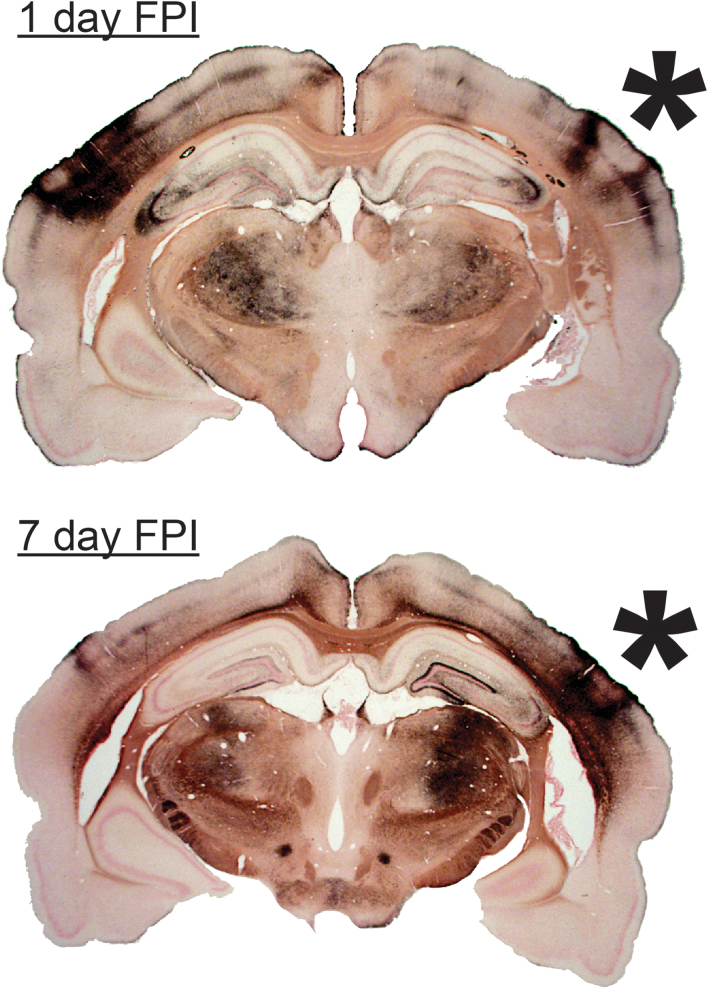
Tissue aligned with the temporal ridge (asterisk) showed increased deposition of argyrophilic reaction product (black) over time post-injury. The mechanical forces of diffuse brain injury reflect off the ventral skull into the temporal ridge, thereby inducing pathology in the lateral nuclei of the thalamus and lateral aspects of the hippocampus. Some sections (right side 1 day DPI) showed tissue spared of argyrophilic reaction at the temporal ridge, suggesting that a non-neural variable may influence the pattern of pathology. FPI, fluid percussion injury.

For all time points post-injury, argyrophilia was evident lateral to midline, under the temporal ridge, and extended the rostral-caudal length of the brain. This length of pathology lies beneath the temporal ridge, with pathology evident bilaterally over the post-injury course. Argyrophilic staining was predominant in the somatosensory cortex (S1BF), lateral portion of the hippocampus (CA3), and ventral posterior thalamus at 1 and 7 DPI (Fig. 5). This pattern of neuropathology occurred systematically across histological sections, with the deepest penetration of pathology in sections associated with the fluid pulse, but not directly under the site of injury induction (center of the craniectomy). In some sections, areas of increased argyrophilic reaction product were inconsistent across the cortex (Fig. 5), suggesting that a variable other than brain tissue properties may influence the pattern of pathology.

### Proposed biomechanical mechanism of rodent traumatic brain injury

**FIG. 6.**
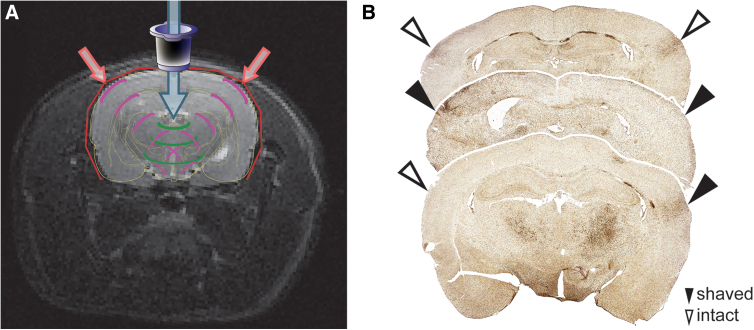
Proposed biomechanical mechanism of rodent TBI. (**A**) Schematic of mechanical forces after midline fluid percussion injury (mFPI) that induce neuropathology. The fluid pulse (blue arrow), generated from the impact of the pendulum on the plunger of a fluid-filled cylinder, lasts only milliseconds and travels through the injury hub into the extradural space, producing mechanical forces that propagate throughout the brain (green arcs). Mechanical forces are then reflected off the ventral portions of the skull and travel throughout the cranium back to the dorsal and lateral portions of the skull (purple arcs). Upon reaching the cortex, increased thickness of the temporal ridge provides a pressure sink or pressure barrier, which ultimately focuses injury-inducing forces on the tissue beneath the temporal ridge, acting as a pinch-point and resulting in observed neuropathology (red arrows). (**B**) To support this proposed mechanism, temporal ridges were shaved uni- or bilaterally to approximate the thickness of the calvarium before injury. Rats then received mFPI and were prepared for Iba-1 immunohistochemistry at 7 DPI to identify areas of neuroinflammation. Rats who received no shaving to the temporal ridge (top brain slice) show focal increase of microglial activation. However, when the temporal ridge was unilaterally (bottom brain slice) or bilaterally (middle brain slice) shaved, an absence of focal neuroinflammation corresponded with the shaved hemisphere(s). Open arrow heads indicate hemispheres with an intact temporal ridge; solid arrow heads indicate hemispheres with a shaved temporal ridge. DPI, days post-injury; Iba-1, ionized calcium-binding adaptor molecule 1; TBI, traumatic brain injury.

Consistent neuropathology occurred beneath and along the temporal ridge after mFPI. We propose a mechanism of injury induced by the mechanical forces of mFPI. Illustrated on a modified coronal MRI section (Fig. 6A), mFPI is initiated (blue arrow) by the fluid pulse and pneumatic forces. This fluid pressure pulse, lasting only milliseconds, then produces mechanical force vectors that propagate throughout the brain (green arcs). Given that the wave propagates through the brain, the force vectors would reflect off the ventral skull, without causing damage. Reflected forces travel dorsal and lateral throughout the cranium, possibly toward the differential thickness of the skull at the temporal ridges (purple arcs). Differential thickness of the skull at the temporal ridges may act as either a pressure sink or a pressure barrier, which ultimately focuses injury-inducing forces back onto the tissue under the temporal ridge. This acts as a “pinch-point” for vulnerable tissue and thereby contributes to the neuropathology observed acutely in the superficial cortical layers (red arrows).

An alternative mechanism of injury would focus extracranial force vectors into the cranium through the temporal ridge, whereby the predicted pathology would initiate at the superficial cortical layers and diffuse ventrally from those points. For either proposed mechanism, the applied forces may remain localized to the originating cerebral hemisphere(s), such that lFPI is lateralized compared to mFPI. We favor the intracranial mechanics proposed mechanism given that pathology shows the largest arc across the cortex at superficial layers.

To support this proposed mechanism, one or two temporal ridge(s) were shaved down before mFPI to approximate the thickness of the calvarium. Rats then received moderate mFPI and were prepared for Iba-1 immunohistochemistry at 7 DPI, the time point with peak neuropathology as identified by de Olmos silver staining. Brains were collected, sectioned, and immunostained with Iba-1 to identify concentrated areas of neuroinflammation, indicative of ongoing neuropathology.^65,70^ Representative histological sections show (Fig. 6B) increased focal neuroinflammation (Iba-1^+^ microglial activation) corresponding to hemispheres with an intact temporal ridge. Rats with a single temporal ridge intact showed an absence of focal neuroinflammation in the hemisphere corresponding with the side of the shaved temporal ridge (Fig. 6B, bottom brain section). Rats with both temporal ridges intact showed the predicted concentrated microglial activation in cortical areas beneath the temporal ridge (Fig. 6B, top brain section). Rats with both of the temporal ridges shaved showed diffuse microglial activation (Fig. 6B, middle brain section).

## Discussion

Here, we present evidence that the skull's shape and thickness help explain the repeatedly documented pathology that occurs lateral to the injury induction site in experimental TBI. A literature search found dozens of articles with figures that showed aspects of neuropathology preferentially localized to tissue beneath the temporal ridge after mFPI and closed skull TBI models. Changes in skull thickness and skull/brain relationship were confirmed using MRI, skulls, and contour imaging of coronal sections from naïve rat skulls. After mFPI, neuropathology primarily occurs in cortical tissue running the rostral-caudal length of the temporal ridge and includes the S1BF, hippocampus, and ventral thalamus in the dorsal-ventral projections of mechanical force. We thus propose a new biomechanical mechanism of experimental TBI mechanical forces to explain this predictable and consistent neuropathology. Further, the uni- or bilateral surgical thinning of the temporal ridge attenuated focal neuroinflammation under the single shaved temporal ridge after mFPI. Together, these data implicate the shape of the rodent skull, in addition to tissue properties, in defining pathology after experimental brain injury.

Since the implementation of FPI, multiple groups have hypothesized the biomechanics of injury based on injury parameters and extent of tissue pathology, rather than localized tissue pathology and the potential contribution of skull shape.^15,71^ As Dixon and colleagues noted, “*fluid moves through the epidural space of the brain after FPI*.”^6^ As shown in Figure 3, the rat skull is not uniform in thickness and could influence mechanical force trajectories applied to the head and skull, with the likely consequence of increased strain and stress on neuronal tissue and tissue forced against different parts of the skull. Recent *in silico* modeling of lFPI included a three-layer hexahedral, element-based skull module with varying Young modulus for each layer.^72^ The model used a uniformly thick skull and predicted significant strain in regions surrounding the initial injury site that dissipate through the brain, in contrast to our neuropathology results and those of others (Table 1).

An opportunity exists for future finite element models to consider differential skull thickness and shape. Also, although each of these studies describes how the fluid pulse interacts with and thus deforms neuronal tissue, they do not explain the unique pattern of neuropathology observed post-injury, nor the forces reflected off the skull that travel through neuronal tissue. As we characterize here, the rat skull is not a uniform thickness and therefore would not absorb or reflect forces equally. The temporal ridge protrudes externally along the rostral-caudal skull axis, potentially increasing bone rigidity. The calvarium, in contact with the dura and brain, is contoured, smooth, and free of protrusions. One hypothesis to explain the temporal ridge relates to the attachment and early use of the muscles of mastication.^73–75^ Suckling and eating after birth can increase strains along the rostral-caudal axis of the rodent skull sufficiently to influence development of the temporal ridge.

In adulthood, the temporal ridge is 75% thicker than the rest of the calvarium, thus providing stiffness along this point of the skull. Undoubtedly, other components of the skull and skeletal development may evolve as the rodent ages. It is not unreasonable to consider that the differences observed in neuropathology between experimental TBI models may be the result of changes to the skull structure as the animal ages, particularly at young ages preceding when the cranial sutures fully fuse. However, the identified literature presents instances of similar pathology across ages and experimental TBI models (Table 1).

Male and female skulls may influence differentially the neuropathology of TBI. Unfortunately, the majority of earlier pre-clinical TBI studies have been performed in male animals, thus limiting the inclusion of sex as a biological variable in the relationship between skull characteristics and neuropathology. Moving forward, inclusion of non-binary sex, non-binary gender, and physiological variables (e.g., hormone cycles) is necessary for a complete understanding of sex-based influence on outcomes post-TBI and may advance personalized medicine.^76^ With regard to skull development, multiple hormones (e.g., estrogen, progesterone, testosterone, thyroxine, and corticosteroids) interact with bone development and remodeling.^77^ Fluctuations in hormone levels acutely (e.g., menstrual cycle, estrous cycle) and over time (e.g., menopause) may influence skull structure and therefore how brain injury forces interact with the skull.^76,78^ Further, sex differences exist in post-injury neuroinflammation, in terms of Iba-1 staining and microglia morphology, over time and across brain regions after midline FPI and controlled cortical impact (CCI).^79,80^ Ultimately, the mechanics of TBI must consider the skull, in addition to the influence of sex, gender, and physiological hormones.

Evidence across the literature indicates that tissue directly underneath the temporal ridge, rather than the impact site, routinely shows TBI-induced neuropathology. We show evidence of this pathology throughout the rostral-caudal extent of coronal sections. This neuropathology, evidenced in multiple histological outcomes by the neurotrauma community, is multi-focal and preferentially in brain regions within proximity to the temporal ridge.^7,12,19,25,39,42,59,81–119^ It is essential to note that the studies listed in Table 1, and Figures 4 and 5, represent decades of multiple neurotrauma laboratories across the world and are the product of numerous surgeons. Thus, the representative neuropathology associated with the temporal ridge is more likely a feature of injury forces in the rodent brain, rather than spurious surgical variation. We contend that the curious neuropathology results from brain tissue forced into the temporal ridge, given that the injury forces reflect off the ventral skull. Transmission through ventral structures and reflection off the ventral skull would predict optic nerve damage, as reported by the Povlishock group, in mFPI.^120,121^

In some cases (Fig. 5), neuropathology is not uniform along the cortex near the temporal ridge, suggesting that tissue may warp non-uniformly at the temporal ridge of the skull, thereby sparing some cortical tissue from the full forces of injury. Aspects of mechanical forces that may damage the brainstem, as has been shown for FPI in the cat, may exist and need further investigation.^122,123^ Further, gyrencephalic brains may absorb or reflect forces in a manner that minimize the influence of an overall thicker skull. We argue that the relationship of the temporal ridge to injured tissue is not coincidental or attributable to differences in surgical technique.

For brain injury models that intentionally penetrate the dura, neuropathology is expected and localized primarily to the impact site. In these focal injury models, cavitation occurs at the injury site, with a penumbra of tissue damage.^124^ However, neuropathology after focal CCI spreads into diffuse pathology of the contralateral hemisphere, with accumulation of argyrophilia in cortical areas under the temporal ridge by 7 DPI.^124^ Alternatively, if the craniotomy for FPI is performed over the temporal ridge, then no overt pathology is observed.^125,126^ Finally, cortical neuropathology in murine diffuse TBI models also traversed the temporal ridge, as presented for mFPI (Supplementary Material), further supporting a role for the temporal ridge in the proceeding neuropathology.

Closed TBI in the rodent results in neuropathology, preferentially in the somatosensory cortex (S1BF), hippocampus (CA3), and ventral thalamus. These three regions lie in a wedge or arc from the ventral midline aspect of the skull base toward the temporal ridge. For our biomechanical model of brain injury forces, these regions may not be uniquely vulnerable to TBI pathophysiology, but rather casualties along the force vectors (Fig. 6A). By removing one temporal ridge, neuroinflammation becomes localized to a single hemisphere, whereas removing both temporal ridges may retain bilateral inflammation because of the geometrical shape of the skull, regardless of the temporal ridges. The authors acknowledge that a study limitation exists by excluding uninjured sham animals with and without a thinned temporal ridge. Although the surgical procedure to thin the skull is unlikely to induce neuroinflammation, tissue damage cannot be ruled out. Additionally, the observed neuroinflammation after modifying the dynamics of injury forces only support, and do not confirm, the interpretation that neuropathology (de Olmos silver stain) is similar to neuroinflammation (Iba-1 immunohistochemistry). A concordance supports both neuropathology and neuro-inflammation after experimental TBI as indicators of localized pathological processes.^64,67–69^

Further, the neuropathology in these regions would predict neurological and behavioral impairments, including somatosensory and cognitive impairment. With regard to the S1BF and ventral lateral thalamus, whisker hypersensitivity and somatosensory dysfunction have been reported.^30–36,67,127–129^ Cognitive performance involving short, long, and working memory, using multiple established mazes, is also impaired after FPI.^11,33,37–47^ These cognitive impairments may involve CA3 processing to perform object pattern completion, cue retrieval in fear conditioning, episodic memory, and spatial memory.^13,130–137^ Thus, the neuropathology associated with the temporal ridge to include the S1BF, CA3, and ventral thalamus manifests with impairments in behavioral performance post-injury.

## Conclusions

By analyzing the curious pathology of mFPI and relating the biomechanical model to other closed TBI models, laboratory studies can take advantage of localized pathology, without overt cavitation, to explore post-traumatic reorganization and repair of the cortex, hippocampus, and ventral thalamus.^65,67^ By focusing on neuronal circuits that regulate somatosensory and cognitive function, investigators may continue to advance our understanding of the disease process that dismantles, repairs, and regenerates circuits in the brain. The pathology of diffuse TBI is the summation of 1) the mechanical forces of the primary injury, 2) the biomechanics of the impacted substrates, 3) the subsequent signaling cascades, and 4) secondary injuries. Ultimately, the acute events of TBI initiate a disease process that leaves persons with debilitating symptoms, which impair their quality of life.^2^ Through continued investigation of the consequences of TBI, we strive to improve the quality of life for our patients by advancing diagnostic techniques and therapeutic interventions.

## Supplementary Material

Supplemental data

## Abbreviations Used

CCI: controlled cortical impact
DPI: days post-injury
FPI: fluid percussion injury
Iba-1: ionized calcium-binding adaptor molecule 1
lFPI: lateral fluid percussion injury
mFPI: midline fluid percussion injury
NIH: National Institutes of Health
MRI: magnetic resonance imaging
RARE: rapid acquisition with relaxation enhancement
RF: radiofrequency
T: Tesla
TBI: traumatic brain injury
